# Genetic deletion or pharmacologic inhibition of the Nlrp3 inflammasome did not ameliorate experimental NASH

**DOI:** 10.1016/j.jlr.2023.100330

**Published:** 2023-01-12

**Authors:** George N. Ioannou, Christian L. Horn, Vishal Kothari, Matthew M. Yeh, Irene Shyu, Sum P. Lee, Christopher E. Savard

**Affiliations:** 1 Division of Gastroenterology, Department of Medicine, Veterans Affairs Puget Sound Health Care System, Seattle, WA, USA; 2 Division of Gastroenterology, Department of Medicine, University of Washington, Seattle WA, USA; 3 Research and Development, Veterans Affairs Puget Sound Health Care System, Seattle, WA, USA; 4 Division of Gastroenterology and Hepatology, Department of Medicine, San Antonio Military Medical Center, Fort Sam Houston, TX, USA; 5 Department of Medicine, Division of Metabolism, Endocrinology and Nutrition, UW Medicine Diabetes Institute, Seattle, WA, USA; 6 Department of Laboratory Medicine and Pathology, University of Washington, Seattle, WA, USA

**Keywords:** cholesterol, dietary fat, inflammation, lipids, liver, fatty liver,, nonalcoholic fatty liver disease, nonalcoholic steatohepatitis,, fibrosis,, innate immune responses, ALP, alkaline phosphatase, ALT, Alanine aminotransferase, AST, Aspartate aminotransferase, DAMP, danger-associated molecular pattern, HFHC, high-fat, high-cholesterol, MCD, methionine-choline deficient, NAFLD, nonalcoholic fatty liver disease, NASH, nonalcoholic steatohepatitis, NLRP3, NLR family pyrin domain containing 3, PAMP, pathogen-associated molecular pattern

## Abstract

It has been postulated that inflammasomes, in particular the NLRP3 (NLR family pyrin domain containing 3) inflammasome, mediate the necroinflammation and fibrosis that characterize nonalcoholic steatohepatitis (NASH) by engaging innate immune responses. We aimed to investigate the impact of genetic deletion or pharmacologic inhibition of the NLRP3 inflammasome on experimental steatohepatitis. Global *Nlrp3* KO (expected to inhibit the NLRP3 inflammasome) or *Casp1* KO (expected to inhibit all inflammasomes) mice were compared to wild type controls after 6 months on a high-fat, high-cholesterol (HFHC, 1% cholesterol) diet known to induce fibrosing steatohepatitis. Additionally, wildtype mice on a HFHC diet (0.75% or 0.5% cholesterol) for 6 months were either treated or not treated with an oral, pharmacologic inhibitor of Nlrp3 (MCC950) that was delivered in the drinking water (0.3 mg/ml). We found that genetic deletion or pharmacologic inhibition of the NLRP3 inflammasome did not ameliorate any of the histological components of fibrosing NASH in HFHC-fed mice. Collectively, these results do not support NLRP3 inhibition as a potential target for human NASH.

Inflammasomes are cytosolic pattern recognition receptors that recognize “exogenous” pathogen-associated molecular patterns (PAMPs) derived from invading pathogens and “endogenous” danger-associated molecular patterns (DAMPs) derived from damaged tissues. Upon activation by their specific PAMPs and DAMPs, inflammasomes form a multiprotein complex containing a receptor protein, an adaptor and an effector, which induces autocleavage of procaspase-1 into active caspase-1 resulting in the maturation and secretion of IL-1b and IL-18 to engage innate immune responses. Through caspase-1 activation, inflammasome activation can also result in pyroptosis, a form of programmed cell death.

The NLRP3 (NLR family pyrin domain containing 3) inflammasome is one of the best-characterized pattern recognition receptors and is well known for its important role in the regulation of immunity and the development and progression of various inflammatory diseases. It has been postulated that the NLRP3 inflammasome is also a key sensor of PAMPs/DAMPs in nonalcoholic fatty liver disease (NAFLD) and a trigger for necroinflammation and fibrosis, resulting in progression to fibrosing nonalcoholic steatohepatitis (NASH) (1). NASH is characterized by lipotoxicity which could result in release of DAMPs from injured hepatocytes, while leaky gut and microbiome dysregulation could result in PAMPs, including lipopolysaccharide, potentially triggering NLRP3 activation. The NLRP3 inflammasome is highly expressed in liver cells involved in the innate immune response, such as the Kupffer Cells but also in hepatocytes. Some prior studies suggested a role for the NLRP3 inflammasome in triggering the necroinflammation that characterizes experimental steatohepatitis in different animal models such as mice fed a methionine-choline deficient (MCD) diet, hyperphagic mice (such the *foz/foz* mice) and LDLR deficient mice on a high-fat diet (2, 3, 4, 5, 6).

Pharmacologic inhibition of Nlrp3 has been reported to have positive results in ameliorating experimental steatohepatitis in *foz/foz* mice on an atherogenic diet (2) or *ApoE−/−* mice on a MCD diet (7). Constitutive activation of the NLRP3 inflammasome causes hepatic inflammation and fibrosis (3, 8). Based on these results, therapeutic targeting of NLRP3 as a treatment of patients with NASH has also been considered, either directly targeting the NLRP3 complex with pharmacologic agents or downstream products including IL-1b and IL-18. Pharmacologic inhibitors of NLRP3, some of which are orally bioavailable, have been recently reviewed (9), including OLT177 (10), MCC950 (2, 11, 12), CY-09 (13, 14) and IFM-514 (7).

We previously demonstrated that C57BL/6J mice on a high-fat, high-cholesterol (HFHC) diet recapitulate the metabolic phenotype (obesity, insulin resistance), laboratory abnormalities and histological features (fibrosing steatohepatitis with perisinusoidal “chicken-wire” fibrosis) that characterize human NASH (15, 16, 17). Furthermore, mice on a HFHC diet demonstrate hepatic necroinflammation and profound hepatic cholesterol loading and cholesterol crystals presenting a plethora of potential PAMPs and DAMPs for NLRP3 activation. Therefore, we investigated whether genetic deletion (using *Nlrp3* KO or *Casp1 KO mice)* or pharmacologic inhibition (using MCC950 a novel oral inhibitor (2, 11, 12)) of the NLRP3 inflammasome would result in amelioration of histological steatohepatitis in mice fed a HFHC diet. Our results demonstrate little or no impact of genetic deletion or pharmacologic inhibition of Nlrp3 on NASH in this animal model and call for a more guarded examination of whether NLRP3 targeting would be a viable therapeutic strategy in human NASH.

## MATERIALS AND METHODS

### Genetic inhibition of *Nlrp3* and *Caspase-1*

**Fig. 1:**
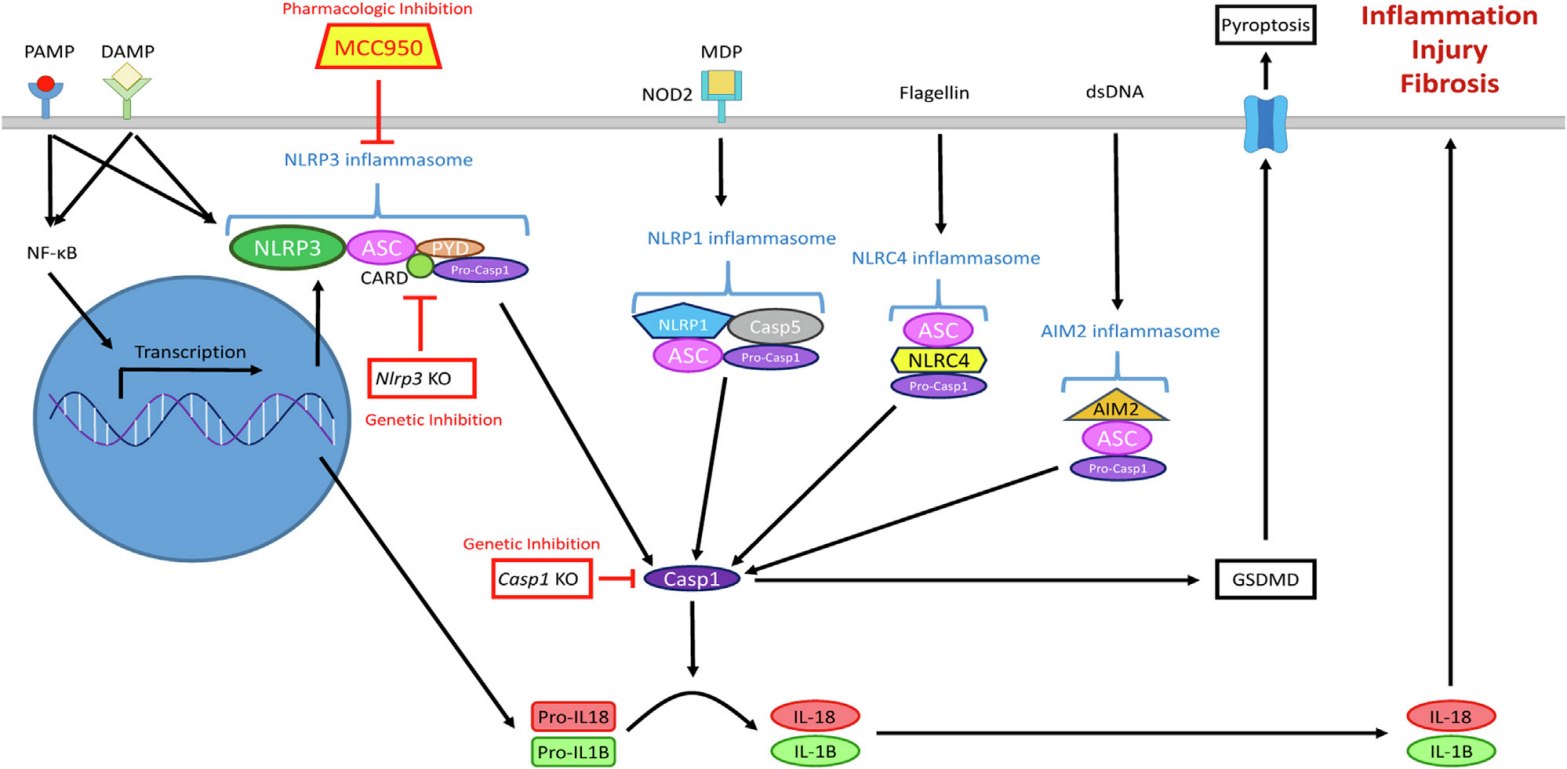
Schematic representation of inflammasome pathways and rationale for the experimental design. Pharmacologic inhibition with MCC950 blocks the Nlrp3 inflammasome. Genetic deletion of *Nlrp3* in the *Nlrp3* KO mice blocks the Nlrp3 inflammasome while genetic deletion of *Casp1* in *Casp1* KO mice blocks all the inflammasomes, since activation of Casp1 is the final common pathway for all inflammasomes. We found that neither pharmacologic inhibition of Nlrp3 with MCC950 nor genetic deletion of Nlrp3 or Casp1 ameliorated any of the histological features of fibrosing NASH in high-fat, high-cholesterol fed mice. NASH, nonalcoholic steatohepatitis.

We employed male mice with a global genetic KO of either the *Nlrp3* gene, targeting only the NLRP3 inflammasome, or the *Caspase-1* gene (*Casp1*), which is the final common pathway for all inflammasomes and therefore results in inhibition of all inflammasomes (Fig. 1).

B6N;129-Nlrp3^tm1Hhf^ mice, with a targeted knock-in of a floxed neomycin cassette into intron 2 of the *Nlrp3* gene, effectively producing a *Nlrp3* expression KO (*Nlrp3* KO) model, and appropriate WT controls of the same strain (C57BL/6N) were obtained from the Jackson Laboratory (Bar Harbor, ME). In addition, B6N.129S2-Casp1^tm1Flv^mice, a *Casp1* KO mouse strain, was also obtained from Jackson Laboratory. WT controls for the *Casp1* KO mice were the same mice used for the *Nlrp3* KO experiment. Starting at 6 months of age, the control and KO mice were given a high-fat (15%, w/w) diet for 6 months supplemented with 1.0% dietary cholesterol purchased from Bio-Serv (Frenchtown, NJ), which has been shown to induce fibrosing steatohepatitis in WT mice (3 groups, n=12 mice/group). Cocoa butter, which contains approximately 60% saturated fat, was the source of the extra fat in these diets (16, 18). Their composition is shown in supplemental Table S1.

### Pharmacologic inhibition of Nlrp3 inflammasome

Starting at 6 months of age, C57BL/6J male mice (Jackson Laboratory, Bar Harbor, ME) were placed on high-fat (15%, w/w) diet supplemented with either 0.75% (24 mice) or 0.5% (18 mice) dietary cholesterol for 6 months. Lower levels of dietary cholesterol (0.75% and 0.5%) were used in this pharmacologic inhibition experiments than in the genetic inhibition experiments (1.0%), because we hypothesized that pharmacologic inhibition might be more likely to ameliorate less severe NASH that occurred at lower dietary cholesterol concentrations—we previously demonstrated a “dose-response” effect of dietary cholesterol (17). During the same 6-month period, half the mice on each diet were given access to MCC950 (an orally available NLRP3-specific inflammasome inhibitor) that was dissolved in the water provided ad libitum to each cage at a concentration of 0.3 mg/ml (12). Dosing of MCC950 via drinking water at this concentration in C57BL/6J mice was shown to be similarly effective in delivering MCC950 into the blood stream (12) as when administered by oral gavage (19) or intraperitoneal injection (11, 20, 21, 22). Fresh diluted MCC950 was added to the water bottles twice per week (changed after 3 or 4 days) for each cage while the control mice received untreated sterile water. Once a month, the water consumption (g fluid/mouse/day respectively) was calculated by measuring the difference in the weight of the water added in the water bottle and the weight of the water remaining 4 days later. This procedure was immediately repeated over another 3-day period. The actual dose of MCC950 ingested by the mice was calculated from the water consumption results (g/mouse/day) times the MCC950 concentration of the water (0.3 mg/ml). There was no difference in the water intake of the mice given MCC950 versus untreated water. Based on the daily intake of MCC950 (g/mouse/day) and the body weight of the mice at each time point, we calculated that the mice ingested an MCC950 dose of 19.1–26.7 mg per Kg/day. This dose is comparable to the MCC950 dose administered in other experiments using gavage or intraperitoneal injection.

### Animal procedures

Mice were housed up to four per cage with unrestricted access to food and water. Mice underwent phlebotomy and were euthanized 6 months after initiation of the experimental diets by cervical dislocation following isoflurane anesthesia, and their livers were harvested as well as subcutaneous and intra-abdominal (epididymal) adipose tissue. All experimental procedures were approved by the Institutional Animal Care and Use Committee of the Veterans Affairs Puget Sound Health Care System.

### Histological assessment of hepatic steatosis, inflammation and fibrosis and cholesterol crystals

Formalin-fixed, paraffin-embedded liver tissue sections were stained with H&E, Masson’s trichrome or Sirius red (for collagen). Histological steatosis, inflammation, and fibrosis were assessed semi-quantitatively using the scoring system of Kleiner *et al.* (23) by consensus of two “blinded”, expert hematopathologists (IS and MY). Sirius red-stained collagen fibers were also quantified using a polarizing microscope by digital image analysis (NIH Image J density software (15)), as the average of 12 random 200x-fields without major blood vessels. Hepatic cholesterol crystals were visualized as we previously described (16, 17, 18, 24, 25) by observing fresh-frozen liver sections under polarized light.

### Hepatic and adipose tissue lipid analysis

Lipids were extracted from frozen mouse liver or adipose tissue (subcutaneous and intra-abdominal) by using the Folch method (26). The neutral lipid fractions were prepared by solid phase extraction, and the triglycerides, cholesterol esters, and free cholesterol were then separated and quantified by normal phase HPLC/Evaporative Light Scattering Detector.

### Other measurements

Blood specimens were collected immediately prior to sacrifice after a 4-h fast and tested for plasma cholesterol, triglycerides, alanine aminotransferase (ALT), aspartate aminotransferase (AST), alkaline phosphatase (ALP), glucose, insulin, and high molecular weight adiponectin. Average food consumption was measured monthly.

To confirm the knockout of *Nlrp3* in the B6N;129-Nlrp3^tm1Hhf^ mice, we measured mRNA expression of the *Nlrp3* gene in liver tissue. Western blot analysis was used to measure protein levels of both the inactive procaspase-1 and active caspase-1 in liver tissue to confirm *Casp1* deletion in B6N.129S2-Casp1^tm1Flv^mice and to confirm adequate inhibition of the Nlrp3 inflammasome by MCC950. We also measured levels of activated Il-1b in plasma using a multiplex assay (Enhanced sensitivity Cytometric Bead Array system, BD Biosciences, Franklin Lakes NJ).

## RESULTS

### Genetic deletion of *Nlrp3* and *Casp1* did not lead to amelioration of fibrosing NASH

**Fig. 2:**
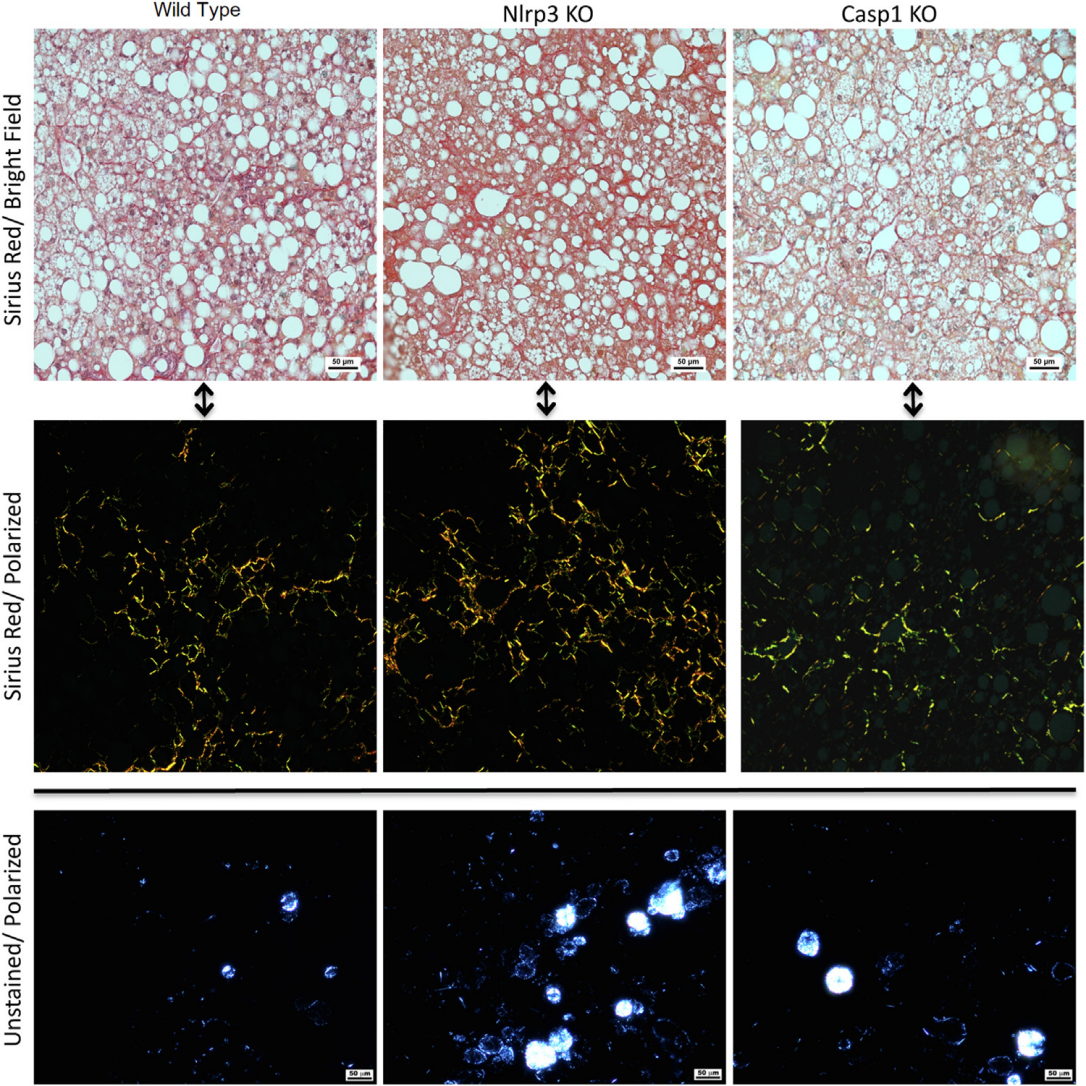
Comparison of hepatic histological features in wild type mice versus *Nlrp3* KO and *Casp1* KO mice after 6 months on a HFHC (1% cholesterol) diet. The wild type mice developed similar grades of steatosis (top row), perisinusoidal fibrosis (middle row), and hepatocyte lipid droplet cholesterol crystallization (lower row) as the *Nlrp3* KO and *Casp1* KO mice. HFHC, high-fat, high-cholesterol.

The WT mice developed obesity, hyperglycemia, elevated serum liver enzymes and hepatic steatosis, inflammation and perisinusoidal fibrosis characteristic of fibrosing NASH, after 6 months on the high fat/ 1% cholesterol diet (Table 1 and Fig. 2), as we previously described (15, 16, 17).

**Table 1:** Comparison of *Nlrp3* KO and *Casp1* KO mice with wild type mice after 6 months on a high-fat/high-cholesterol (HFHC) diet

Genetic Strain	Wild Type	*Nlrp3* KO	*Casp1* KO
Genetic background	C57BL/6N	C57BL/6N	C57BL/6N
Diet	HFHC 1.0% Cholesterol	HFHC 1.0% Cholesterol	HFHC 1.0% Cholesterol
Number of mice	12	10	10
Body weight (g)	46.2 ± 1.9	50.6 ± 4.4^b^	45.6 ± 2.9
Liver weight (g)	4.3 ± 0.4	5.3 ± 1.0^b^	3.8 ± 0.5^b^
Liver weight/body weight (%)	9.4 ± 0.7	10.5 ± 1.2^b^	8.2 ± 0.7^a^
Food consumption (g/mouse/day)	2.9 ± 0.1	3.3 ± 0.2^a^	2.5 ± 0.2^a^
Plasma levels (fasting)			
ALT (U/L)	382 ± 104.5	424.7 ± 163.1	391.6 ± 142.1
AST (U/L)	276.9 ± 78.4	309.0 ± 87.1	291.6 ± 117.2
ALP (U/L)	105.3 ± 17.9	147.0 ± 43.2^b^	104.9 ± 25.9
Cholesterol (mg/dl)	303.1 ± 40.7	344.6 ± 59.0	328.4 ± 56.3
Triglyceride (mg/dl)	62.9 ± 12.7	69.3 ± 10.8	69.3 ± 13.3
Glucose (mg/dl)	348.1 ± 84.6	316.4 ± 39.0	377.0 ± 38.1
Insulin (ng/ml)	2.01 ± 1.33	4.93 ± 3.92	1.76 ± 1.22
HOMA-IR	37.9 ± 25.5	82.2 ± 62.5	33.9 ± 22.3
HMW-adiponectin (mg/ml)	1.49 ± 0.34	1.61 ± 0.67	2.16 ± 0.23^b^
Hepatic histology, n (%)			
Steatosis grade			
0	0 (0%)	0 (0%)	0 (0%)
1	0 (0%)	0 (0%)	0 (0%)
2	0 (0%)	0 (0%)	0 (0%)
3	12 (100%)	10 (100%)	10 (100%)
Inflammation grade			
0	0 (0%)	0 (0%)	0 (0%)
1	0 (0%)	0 (0%)	1 (10%)
2	11 (92%)	6 (60%)	6 (60%)
3	1 (8%)	4 (40%)	3 (30%)
Fibrosis stage			
0	2 (17%)	0 (0%)	5 (50%)
1	10 (83%)	9 (90%)	5 (50%)
2	0 (0%)	1 (10%)	0 (0%)
3	0 (0%)	0 (0%)	0 (0%)
Sirius red staining (fibrosis), % area	1.00 ± 0.64	2.00 ± 1.92	1.19 ± 0.70
Hepatic cholesterol birefringence	Very common	Very common	Very common
Hepatic lipids (mg/ g liver)			
Cholesterol ester	78.5 ± 32.6	122.9 ± 42.3^a^	106.2 ± 28.0^b^
Free cholesterol	0.52 ± 0.47	0.62 ± 0.31	1.30 ± 0.45^a^
Triacylglycerides	345.5 ± 72.2	295.2 ± 69.8	426.3 ± 67.3^b^
Diacylglycerides	1.34 ± 0.27	1.46 ± 0.3	1.41 ± 0.25
Epididymal fat (mg/ g fat)			
Cholesterol ester	0.52 ± 0.22	0.43 ± 0.14	0.39 ± 0.13
Free cholesterol	3.19 ± 1.12	3.48 ± 0.91	2.03 ± 0.44^b^
Subcutaneous fat (mg/ g fat)			
Cholesterol ester	0.23 ± 0.09	0.20 ± 0.09	0.16 ± 0.06
Free cholesterol	1.64 ± 0.83	1.51 ± 0.39	1.42 ± 0.34

ALT, alanine aminotransferase; AST, aspartate aminotransferase; ALP, alkaline phosphatase; HOMA, homeostasis model assessment; HFHC, high-fat, high-cholesterol; HMV, high molecular weight.

a *t* test *P* < 0.01 compared to wild type.

b *t* test *P* < 0.05 compared to wild type.

The *Nlrp3* KO mice on the same diet had slightly higher body weight, liver weight, liver weight/body weight ratio, and average food consumption. The *Nlrp3 KO* mice also developed fibrosing NASH with similar elevated levels of plasma ALT, AST, and ALP compared to the WT mice, similar levels of hepatic steatosis, and slightly higher levels of hepatic inflammation grade and hepatic fibrosis by histological staging and quantitative Sirius red staining—a difference that was not statistically significant (Table 1). Hepatic levels of cholesterol ester were higher in the *Nlrp3* KO mice than WT mice, with no difference in hepatic free cholesterol or triacylglyceride levels. Hepatic cholesterol crystals were equally prominent in frozen liver sections of both *Nlrp3* KO and WT mice (Table 1 and Fig. 2).

The free cholesterol and cholesterol ester contents of the epididymal and subcutaneous fat were not different between *Nlrp3* KO and WT mice (Table 1).

The *Casp1* KO mice also developed fibrosing NASH and compared to WT mice, they had similar body weight, liver weight/body weight ratio, average food consumption, plasma levels of ALT, AST and ALP, and hepatic steatosis, inflammation and fibrosis measured by Sirius red.

### Pharmacologic inhibition of NLRP3 inflammasome with MCC950 did not lead to amelioration of fibrosing NASH

**Fig. 3:**
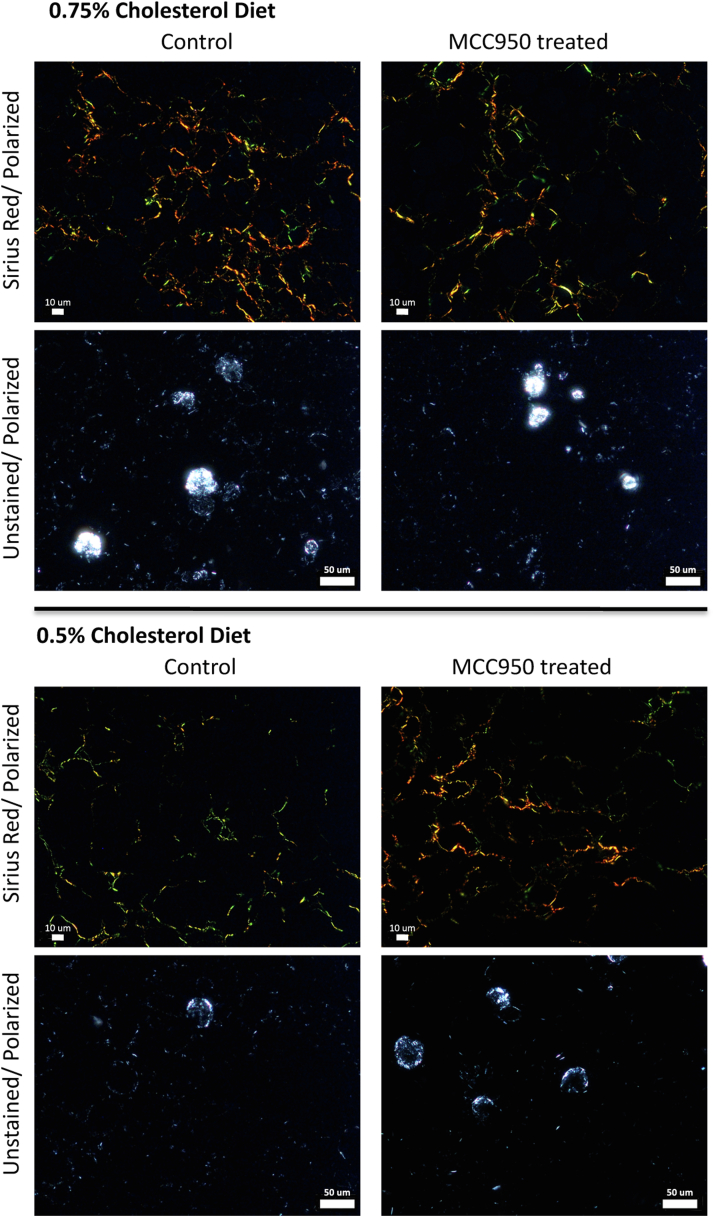
Comparison of wild type mice on HFHC diet (0.75% or 0.5% cholesterol) that were either treated with MCC950 in their drinking water or not. There was little difference in MCC950 treated versus Control mice in the degree of hepatic fibrosis (Sirius Red staining) or cholesterol crystallization in hepatocyte lipid droplets (unstained/polarized light) for both 0.75% and 0.5% cholesterol diets HFHC, high-fat, high-cholesterol.

Mice on a 0.75% cholesterol diet that did not receive MCC950 developed fibrosing NASH, albeit with lower levels of fibrosis by histological staging or quantitative Sirius red staining than the mice treated with 1% cholesterol in the genetic deletion experiments described above. Compared to mice on a 0.75% cholesterol diet that were not treated with MCC950, those that were treated with MCC950 in their drinking water had lower liver weight and plasma cholesterol, similar levels of plasma ALT, AST and ALP, similar levels of hepatic steatosis and inflammation and slightly lower levels of hepatic fibrosis by quantitative Sirius red staining or by histological stage that were not statistically significant (Table 2 and Fig. 3).

**Table 2:** Comparison of MCC950-treated^a^ mice to untreated mice fed a HFHC diet for 6 months

Genetic Background	C57BL/6J	C57BL/6J	C57BL/6J	C57BL/6J
Diet	HFHC 0.75% Cholesterol	HFHC 0.75% Cholesterol	HFHC 0.5% Cholesterol	HFHC 0.5% Cholesterol
MCC950 treatment	No	Yes	No	Yes
Number of mice	11	10	9	8
Body weight (g)	48.7± 4.0	45.9 ± 4.2	43.5 ± 5.0	44.9 ± 7.9
Liver weight (g)	4.4 ± 0.9	3.6 ± 0.9^b^	2.8 ± 1.2	3.2 ± 1.7
Liver weight/body weight (%)	9.0 ± 1.0	7.8 ± 1.3^b^	6.2 ± 2.2	6.8 ± 2.6
Food consumption (g/mouse/day)	3.1 ± 0.3	3.1 ± 0.4	3.0 ± 0.3	3.0 ± 0.4
Fluid consumption (g/mouse/day)	2.8 ± 0.2	2.9 ± 0.3	3.0 ± 0.3	3.1 ± 0.4
MCC950 dose (mg/Kg/ day)	None	19.2 ± 1.9	None	20.7± 2.9
Plasma levels (fasting)				
ALT (U/L)	365.3 ± 57.1	335.1 ± 122.7	171.3 ± 215.9	186.0 ± 203.8
AST (U/L)	261.7 ± 44.4	269.4 ± 156.1	139.3 ± 135.3	146.2 ± 128.6
ALP (U/L)	216.9 ± 30.1	199.0 ± 53.4	126.4 ± 50.1	140.5 ± 65.0
Cholesterol (mg/dl)	323.1 ± 38.5	232.5 ± 93.3^c^	196.9 ± 58.1	205.2 ± 117.8
Triglyceride (mg/dl)	43.7 ± 9.1	38.5 ± 9.5	46.0 ± 13.9	44.8 ± 19.1
Glucose (mg/dl)	293.9 ± 33.3	248.8 ± 44.7^b^	289.2 ± 49.6	304.0 ± 28.2
Hepatic histology, n (%)				
Steatosis grade				
0	0 (0%)	0 (0%)	2 (22%)	2 (25%)
1	0 (0%)	0 (0%)	0 (0%)	0 (0%)
2	0 (0%)	0 (0%)	0 (0%)	0 (0%)
3	11 (100%)	10 (100%)	7 (78%)	6 (75%)
Inflammation grade				
0	0 (0%)	0 (0%)	0 (0%)	0 (0%)
1	0 (0%)	0 (0%)	0 (0%)	2 (25%)
2	0 (0%)	0 (0%)	4 (44%)	1 (12%)
3	11 (100%)	10 (100%)	5 (56%)	5 (63%)
Fibrosis stage				
0	4 (36%)	7 (70%)	2 (22%)/	2 (25%)
1	7 (64%)	3 (30%)	7 (78%)	5 (63%)
2	0 (0%)	0 (0%)	0 (0%)	1 (12%)
3	0 (0%)	0 (0%)	0 (0%)	0 (0%)
Sirius red staining (fibrosis), % area	0.37 ± 0.44	0.17 ± 0.12	0.16 ± 0.14	0.31 ± 0.35
Hepatic cholesterol crystals	Very common	Very common	Uncommon/ widely scattered	Uncommon/ widely scattered
Hepatic lipids (mg/ g liver)				
Cholesterol ester	72.1 ± 13.3	52.0 ± 14.7^c^	19.1 ± 15.2	26.6 ± 31.4
Free cholesterol	1.17 ± 0.60	0.78 ± 0.22	0.57 ± 0.49	0.45 ± 0.36
Triacylglycerides	108.2 ± 36.2	110.4 ± 24.4	122.4 ± 42.3	76.1 ± 48.4
Diacylglycerides	0.35 ± 0.29	0.27 ± 0.09	0.15 ± 0.07	0.21 ± 0.14

ALT, alanine aminotransferase; AST, aspartate aminotransferase; ALP, alkaline phosphatase; HFHC, high-fat, high-cholesterol.

a MCC950 was dissolved in the drinking water of the mice at a concentration of 0.3 mg/ml.

b *t*-test *P* < 0.05 compared to No MCC950 group.

c *t*-test *P* < 0.01 compared to No MCC950 group.

Mice fed a high fat diet with 0.5% cholesterol generally had lower levels of serum AST, ALT and ALP, and lower levels of steatosis, inflammation, or quantitative fibrosis than mice fed 0.75% cholesterol (Table 2 and Fig. 3). Among mice on a 0.5% cholesterol, there were no differences between those that received MCC950 and those that did not in all parameters measured, including levels of plasma AST, ALT and ALP, hepatic lipid composition and hepatic steatosis, inflammation, and fibrosis.

### Confirmation of NLRP3 inflammasome inhibition in our genetic and pharmacologic experimental models

We confirmed the knockout of *Nlrp3* in the B6N;129-Nlrp3^tm1Hhf^ mice by measuring mRNA expression of the *Nlrp3* gene in liver tissue, which showed no detectable mRNA in the KO mouse livers compared to high expression levels in the WT mice.

We measured procaspase-1 and active-caspase-1 protein levels by Western blot in liver tissue (supplemental Fig. S1). WT mice treated with water (Control) in our pharmacologic inhibition experiments demonstrated a band for both procaspase-1 and active caspase-1 as expected.

WT mice treated with MCC950, the pharmacologic inhibitor of Nlrp3, demonstrated a band for procaspase-1, but no band for active-caspase-1, confirming that MCC950 truly inhibited the Nrlp3 inflammasome and prevented conversion of procaspase-1 to active-caspase-1. *Casp1* KO mice demonstrated no band for either procaspase-1 or active caspase-1, confirming that these mice were true KOs.

Plasma Il-1b levels were extremely low with high variability between samples which precluded us from demonstrating any statistically significant differences between the groups, as shown in supplemental Fig. S2, except for the *Nlrp3* KO mice, which had significantly lower levels as expected.

## DISCUSSION

We found that genetic deletion of *Nlrp3* or *Casp1*, or pharmacologic inhibition of Nlrp3 using an oral inhibitor (MCC950) did not ameliorate the development of any of the histological features of fibrosing steatohepatitis (hepatic fibrosis, inflammation, or steatosis) in HFHC-fed mice. In contrast to prior experiments, our results temper the enthusiasm for Nlrp3 inhibition as a therapeutic strategy in human NASH.

There are no approved pharmacotherapies for NASH. The NLRP3 inflammasome has been suggested as an attractive potential target for NASH therapies for a number of reasons. First, the NLRP3 inflammasome has been shown to be activated in some mouse models of NASH. Second, inflammasome activation results in caspase-1 cleavage and activation, which in turn causes activation of pro-inflammatory mediators IL-1b and IL-18 as well as inducing pyroptosis, all factors thought to be relevant in NASH pathogenesis. Third, candidate PAMPs and DAMPs that can potentially initiate NLRP3 activation are prominent in NASH. Indeed, prior studies in animal models suggested that inhibition of Nlrp3 ameliorated some histologic components of NASH whereas constitutive activation of Nlrp3 exacerbated NASH features (2, 3, 4, 5, 6, 7, 8, 27). Given this promising potential of Nlrp3 inhibition as a treatment for NASH, we undertook a systematic investigation of the effects of both genetic deletion and pharmacologic inhibition of Nlrp3 on experimental steatohepatitis. We used an animal model of NASH, the HFHC-fed mouse, that provides a plethora of potential DAMPs (including cholesterol crystals) known to activate Nlpr3 as well as recapitulating the histologic and metabolic phenotype of chronic NASH. However, we found that neither genetic deletion nor pharmacologic inhibition of Nlrp3 ameliorated any of the histologic features of NASH. Furthermore, in contrast to some prior studies (6, 28), we found that genetic deletion of *Casp1*, the final common pathway for all inflammasomes, did not result in improved NASH histology. Differences between our findings and prior studies are likely due to differences in the mouse models of NASH that were employed in each study, and only future human studies can resolve whether inhibiting Nlrp3 specifically or all inflammasomes via caspase-1 inhibition can improve human NASH histology—which would also indirectly suggest which experimental design reflects the human condition most closely.

In a prior study (2), MCC950 was gavaged daily 5 days/week (20 mg/kg/day, in saline) for 8 weeks in female foz/foz mice (which are hyperphagic due to a mutation in the Alms1 gene and develop severe obesity and hyperglycemia) following 16 weeks on an atherogenic diet. In that study, the MCC950-treated mice were less likely to develop steatosis, inflammation or “definite NASH” and had less fibrosis than the untreated mice. It is unclear why MCC950 administration did not result in a similar amelioration of steatosis, inflammation, and fibrosis in our experiments. We administered MCC950 in the drinking water, rather than by gavage, however the average daily dose ingested was almost identical (19.2 and 20.7 mg/Kg/day) to the dose gavaged in the prior study and furthermore in our experiments mice were treated with MCC950 every day (rather than only 5 days/week) and for the entire duration of the HFHC feeding (6 months) rather than only for 8 weeks after the steatohepatitis had already developed in the prior study. The bioavailability of MCC950 administered to mice in the drinking water at 0.3 mg/ml and its effectiveness at inhibiting the NLRP3 inflammasome in vivo was established in a prior published study (12) and this dosage was recommended to us by the manufacturers of MCC950 (personal communication with Dr Avril Robertson). In another recent study, administration of a different Nlrp3 inhibitor called IFM-514 via intra-peritoneal injection (100 mg/kg) daily, 5 days/week for 4 weeks to ApoE^−/−^ mice on a MCD diet was also found to reduce hepatic steatosis, inflammation, and fibrosis (7). However, when IFM-514 was injected into ApoE^−/−^ mice on a Western diet, it did not reduce inflammation or fibrosis (7). A different, recently described Nlrp3-specific inhibitor called CY-09 injected daily intraperitoneally in WT mice on a high-fat diet for 14 weeks resulted in less weight gain and hepatic steatosis (hepatic inflammation and fibrosis were not reported) (13). Although it is impossible to reconcile all these diverse experimental results, we suspect that the type, dose and mode of administration of the Nlrp3 inhibitor and the age, duration, genetic profile, diet, and type of lipotoxic injury of the experimental NASH mouse model used all likely affect the observed results. Given conflicting animal model results, it is difficult to predict what the effect of Nlrp3 inhibition in human NASH would be and ultimately clinical trials would be needed to establish this.

Some noteworthy features of the HFHC-fed mouse model and experimental design that we employed are worth highlighting as they may relate to human NASH. We started our HFHC diets in 6-month-old mice and continued these NASH-inducing diets for another 6 months—as compared to other experimental designs that use much younger mice and shorter interventions. We believe this is more analogous to human, adult NASH which develops most commonly over many years in middle or advanced age. We used nongenetically modified mice for the Nlrp3 inhibition experiments, since human NASH is not driven predominantly or exclusively by a particular genetic polymorphism. C57BL/6J mice on a HFHC diet for at least 6 months develop profound steatosis, necroinflammation and also perisinusoidal fibrosis, which is particularly important because fibrosis is now recognized as the main driver of pathology and adverse clinical outcomes in human NASH and hence treatments for NASH should be targeting fibrosis resolution. On the other hand, C57BL/6J mice, which we used in our MCC950 pharmacologic Nlrp3 inhibition experiments, have a functional defect in NLRP12 (29) which could complicate the interpretation of our results. We also acknowledge that the B6N.129S2-Casp1^tm1Flv^mice which we employed as a *Casp1* KO, also has a known loss in caspase-11 gene expression (30). Caspase-11 is involved in a noncanonical inflammasome pathway. However, loss of both caspase 1 and caspase-11 function in B6N.129S2-Casp1^tm1Flv^mice failed to ameliorate histological NASH.

The fact that neither Nlrp3 deletion/pharmacologic inhibition nor Casp1 deletion (which blocks the final common pathway for all inflammasomes) ameliorated any of the histologic features of NASH, suggests that at least in the HFHC-fed mouse model these features are not driven predominantly by inflammasome activation. It is possible that other pattern-recognition receptors are involved in sensing DAMPs which are exposed or released upon cell death under “sterile” conditions of lipotoxicity. Such pattern-recognition receptor candidates might include transmembrane proteins such as the toll-like receptors (31) and C-type lectin receptors (shown to respond to crystalline structures such as uric acid crystals (32)) as well as cytoplasmic proteins such as retinoic acid-inducible gene-I-like receptors (33).

In conclusion, although NLRP3 activation can induce hepatic inflammation and fibrosis, inhibiting the NLRP3 pathway does not ameliorate any of the histological features of NASH in an experimental mouse model suggesting that they are not mediated by NLRP3 inflammation.

## Data availability

All the data described in the article are located within the article including the supplemental data.

## Supplemental data

This article contains supplemental data (15, 16).

## Conflict of interest

The authors declare that they have no known competing financial interests or personal relationships that could have appeared to influence the work reported in this paper.
