# Supplementary material for: Genetic deletion or pharmacologic inhibition of the Nlrp3 inflammasome did not ameliorate experimental NASH
Source: J Lipid Res. 2023 Jan 12;64(2):100330. doi: 10.1016/j.jlr.2023.100330 (PMC9944495; doi:10.1016/j.jlr.2023.100330)
Supplement: Supplemental tables and figures [file mmc1.docx]

**Supplemental Table 1.** Composition of the high-fat, high-cholesterol diet[1, 2]

| Protein, g/100g | 13 |
| --- | --- |
| Carbohydrate, g/100g | 56 |
| Fat, g/100g | 15 |
| Cholesterol, g/100g | 0.5%, 0.75% or 1.0% |
|  |  |
| Fatty Acids, g/100g |  |
| C14:0 | 0.013 |
| C16:0 | 3.6 |
| C16:1 | 0.03 |
| C18:0 | 4.5 |
| C18:1 | 4.8 |
| C18:2 | 1.4 |
| C18:3 | 0.1 |
| Total Saturated | 8.2 |
| Total Monounsaturated | 4.8 |
| Total Polyunsaturated | 1.5 |
|  |  |
| Protein, kcal/gm | 0.5 |
| Carbohydrate, kcal/gm | 2.3 |
| Fat, kcal/gm | 1.4 |
|  |  |
| Total kcal/gm | 4.2 |

**Supplemental Figure 1. Pro-Caspase-1 and Active-Caspase-1 Western Blot Analysis in Liver Tissue**

Wild-type mice treated with water (Control) in our pharmacologic inhibition experiments demonstrated a band for both pro-caspase-1 and active caspase-1 as expected.

Wild-type mice treated with MCC950, the pharmacologic inhibitor of Nlrp3, demonstrated a band for pro-caspase-1 but no band for active-caspase-1, confirming that MCC950 truly inhibited the Nrlp3 inflammasome and prevented conversion of pro-caspase-1 to active-caspase-1. *Casp1* knockout (KO) mice demonstrated no band for either pro-caspase-1 or active caspase-1, confirming that these mice were true knockouts.


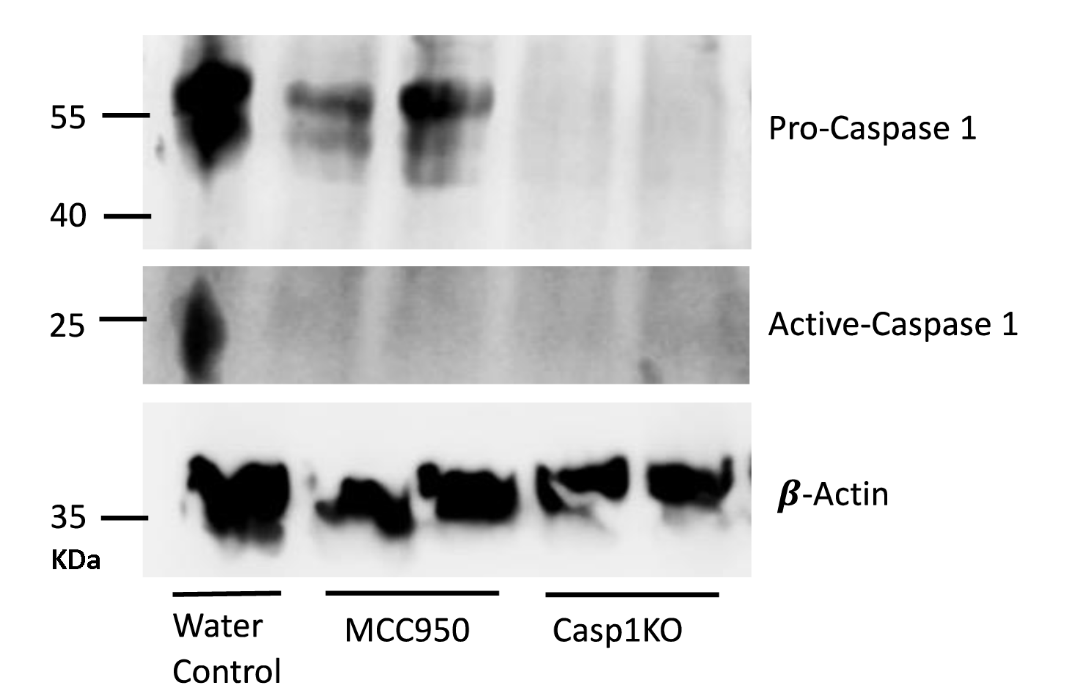


**Supplemental Figure 2. Activated Il-1b in plasma measured by multiplex assay**

We measured levels of activated Il-1b in plasma using a multiplex assay. These levels were extremely low with high variability between samples which precluded us from demonstrating any statistically significant differences between the groups, as shown in **Supplemental Figure 2**, except for the Nlrp3 KO mice, which had significantly lower levels as expected.


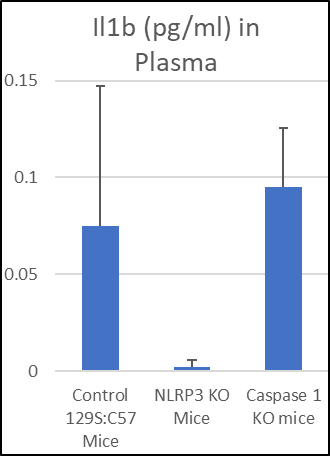

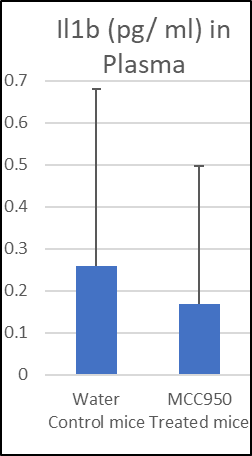


[1] Ioannou GN, Haigh WG, Thorning D, Savard C. Hepatic cholesterol crystals and crown-like structures distinguish NASH from simple steatosis. Journal of lipid research 2013;54:1326-1334.

[2] Savard C, Tartaglione EV, Kuver R, Haigh WG, Farrell GC, Subramanian S, et al. Synergistic interaction of dietary cholesterol and dietary fat in inducing experimental steatohepatitis. Hepatology 2013;57:81-92.
